# Understanding public perceptions in social media responses to posts about acute severe hepatitis of unknown etiology in Indonesia: a qualitative study

**DOI:** 10.1186/s12879-023-08195-y

**Published:** 2023-05-08

**Authors:** Gregorius Abanit Asa, Nelsensius Klau Fauk, Hailay Abrha Gesesew, Kristen Marie Foley, Belinda Lunnay, Paul Russell Ward

**Affiliations:** 1 Research Centre for Public Health, Equity and Human Flourishing (PHEHF), Torrens University Australia, Adelaide, South Australia Australia; 2 Sanggar Belajar Alternatif (SALT), Atambua, Nusa Tenggara Timur Indonesia; 3 Institute of Resource Governance and Social Change, Kupang, Indonesia; 4 College of Health Sciences, Mekelle University, Mekelle, Tigray, Ethiopia

**Keywords:** Acute severe hepatitis of unknown etiology, Social media, Disease prevention, Indonesia

## Abstract

**Background:**

Acute Severe Hepatitis of Unknown Etiology (ASHUE) emerged as a new global outbreak in Indonesia early May 2022, coinciding with the COVID-19 pandemic. This study aimed to understand public reactions and responses to the emergence of ASHUE Indonesia and to Government-led disease prevention responses. Understanding how the public perceived government-led preventive messaging about the hepatitis outbreak is crucial to controlling viral spread – particularly given the rapid and unforeseen emergence of ASHUE coincided with COVID-19 and public trust in the Indonesian Government to manage health outbreaks was already tenuous.

**Methods:**

Social media users’ responses to information disseminated via Facebook, YouTube, and Twitter were analyzed to understand public perceptions about ASHUE outbreak and their attitudes toward Government-led prevention measures. Data were extracted on a daily basis from 1st May 2022 to 30th May 2022 and analyzed manually. We inductively generated the codes, from which we formed a construct and then grouped to identify themes.

**Results:**

A total of 137 response comments collected from 3 social medial platforms were analyzed. Of these, 64 were from Facebook, 57 were from YouTube, and 16 were from Twitter. We identified 5 main themes, including (1) disbelief in the existence of the infection; (2) suspicion about a potential new business after COVID-19; (3) suspicion that COVID-19 vaccine(s) are the cause; (4) religion-related fatalism and (5) trust in government measures.

**Conclusions:**

The findings advance knowledge about public perceptions, reactions and attitudes towards the emergence of ASHUE and the efficacy of disease countermeasures. The knowledge from this study will provide an understanding of why disease prevention measures might not be followed. It can be used to develop public awareness programs in Indonesia about both the ASHUE and its possible consequences and the available healthcare support.

## Introduction

ASHUE first emerged in children in the United Kingdom (UK) of Great Britain and Northern Ireland in early April 2022 [1]. The disease spread quickly, prefacing a global outbreak. As of 5th April 2022, 10 cases of severe acute hepatitis in children aged under 10 years in Scotland were confirmed by the World Health Organization (WHO), and another 74 cases were reported in the UK on 8th April 2022 [1]. The disease was then reported to have spread to 11 European countries with a total of 169 cases as of 21st April 2022 [2], and to countries outside the European continent, including Israel, Argentina, Serbia, Japan, and Indonesia [3]. As of 13th May 2022, more than 450 cases and 11 deaths were reported to be linked to the mysterious hepatitis outbreak globally [4]. In Indonesia, the first ASHUE case was announced by the Indonesian Health Ministry on 1st May 2022 and as of 24th May 2022, there were 16 cases in children (including 3 deaths) reported in different provinces [3, 4].

The general symptoms of the infection are diarrhea, jaundice, vomiting, abdominal pain, and in some cases, acute liver failure [5, 6]. The WHO categorized ASHUE cases as either confirmed (not applicable at present), probable (“person presenting with an acute hepatitis (non hepA-E*) with serum transaminase > 500 IU/L (AST or ALT), who is 16 years and younger, since 1 October 2021”), and epi-linked cases (“a person presenting with an acute hepatitis (non hepA-E*) of any age who is a close contact of a probable case, since 1 October 2021”) [1, 3]. Although the disease was detected in April 2022, it is thought to have existed since January and March 2022 [2]. Its cause, however, has remained a global question, a ‘mystery’ - hence it is named ASHUE [2]. Hepatitis viruses (A, B, C, D, and E) are not reported to be the causes of ASHUE [5]. A study in the UK found that adenovirus was detected in 72% of children with acute severe hepatitis, and adenovirus 41 F was found in 18 subtyped cases [5, 7], which is reported as an uncommon subtype and generally occurs in immunocompromised patients and young children with compromised immune systems [5]. However, adenovirus has not been proven to be the cause of ASHUE [5, 8]. Similarly, the link between SARS-CoV-2 infection and acute severe hepatitis is unproven [8]. Further studies in countries including more details on clinical and exposure histories, microbiological tests, and toxicology tests have been requested by the WHO to determine its causes [2] .

General risk mitigation and infection control strategies to prevent the spread of ASHUE have been disseminated through social media. Similarly, the Government of Indonesia has disseminated evidence-based preventive measures including washing hands, drinking water that has been pre-boiled, disposing used diapers in sanitary ways, keeping physical distance from other people, wearing masks when in contact with other people, and encouraging parents to report if their children are experiencing symptoms consistent with ASHUE [9]. However, media news coverage on the emergence of ASHUE has been accompanied by many debates in Indonesia – perhaps following the contentious nature of COVID-19 vaccine development. In order to achieve disease control at a population level, it is crucial that the knowledge from this study presented in the paper will assist to know why preventive measures tend not to be followed. We have observed a divide between followers of Government advice and those for whom uptake is delayed or withheld completely.

It is therefore crucial to explore public perceptions about the outbreak and this is possible by analyzing the content of Indonesian Government disease prevention messaging through one medium which is social media where the public has an opportunity to comment/respond. Such an analysis provides insight into public perceptions about the disease including its causation and etiology as well as attitudes towards preventive measures. To our knowledge only a few studies in the US and Europe [5, 8] have been conducted on this topic. The objective of the study is to understand the Indonesians’ perceptions posted on social media sites about the emergence of ASHUE. This is the first qualitative study exploring public perceptions in responses to Indonesian government social media posts about ASHUE and public attitudes to preventive health measures recommended for containing it. Knowledge gained from this study will illuminate potential barriers and facilitators to awareness, understanding, and public action on this currently emerging ‘mysterious’ hepatitis outbreak.

## Methods

### Study design and participants

A qualitative study was designed to explore Indonesians’ perceptions about ASHUE by analyzing Indonesian social media users’ comments on Facebook, YouTube and Twitter. Participants we term ‘social media users’ were those who commented on content posted on social media sites related to ASHUE.

### Data collection

Social media posts from three platforms: Facebook, YouTube and Twitter, were collected for analysis from 1st May 2022 - when the Indonesian Government announced the outbreak - until 30th May 2022, when there was no new additional information being posted by the Government to social media users. Data were Indonesian social media users’ direct responses to posts made from Indonesian Health Department accounts such as Twitter @KemenkesRI and Facebook *Kementerian Kesehatan RI*, Government advice disseminated via other social media and general news about ASHUE posted on YouTube. From 5th May 2022 to 30th May 2022, we extracted daily relevant samples of Indonesian-only posts related to ASHUE. The hashtags #misteriushepatitis and #akuthepatitis were used to identify posts related to ASHUE. Although we tried to capture a variety of social media posts, our main focus was on the depth of the comments about ASHUE in Indonesia and the Indonesian Government’s social media posts about the infection (for understanding public perceptions, reactions) made by social media users. Therefore, the sample size in this qualitative study was determined by the ‘richness’ of comments made on posts on the 3 social media platforms. We stopped extracting data once we perceived there to be a saturation of meaning within our coding framework.

Instagram was not included as a source, because although the Government used this platform to post content concerning ASHUE, there seemed to be limited discussion/debate from social media users on the content, perhaps because this form of social media operates using agreement binaries (i.e. like or dislike functions) and users are less likely to make comments. The three platforms selected (Facebook, YouTube and Twitter) were selected because of their popularity within the Indonesian population. The number of active social media users in Indonesia was reported to reach 191 million (about 68% of total population) [10]. Of this, 176 million were active Facebook users [11], 130 million YouTube users, and 18 million Twitter users [12]. Although the number of Twitter users is much lower than the other platforms, it is globally used for tracking news trends of all descriptions including health. Abbreviations and emojis were deleted. The inclusion and exclusion criteria are outlined in Table 1.

**Table 1:** Eligible criteria to recruit data fragments

Inclusion criteria	Exclusion criteria
Comments related to ASHUE on government press release, health advice from health practitioners and preventive measures suggested either by government or by public, and general news about ASHUE	Comments not related to ASHUE
Comments include ASHUE and COVID-19.	Comments related to COVID-19 only
Comments were at least one sentence or several sentences long	Only one or two words in comments (e.g. not believe, fake news, new business, do not trust)

### Data analysis

The analysis was conducted in Indonesian and people’s comments were translated into English for the purposes of reporting results here. We ensured the accuracy of the translation by performed an iterative process of checking and re-checking original comments and translations to examine the meaning in both languages [13]. Data were analyzed manually. Social media comments (reactions/responses) relevant to the study purpose were extracted to a text document and were highlighted, commented and labelled in order to identify ideas, concepts and perceptions for analysis. The content of public responses to posts was then inductively and individually coded. Codes that formed a construct were then grouped to identify themes. The identified themes were reviewed and discussed by all authors to ensure interpretive rigour, and this involved discussing data coded at a construct within each theme to check for agreement [14, 15].

## Results

In total, we captured 308 comments and 171 comments were removed. A total of 137 comments were analyzed from the three social media platforms. Of these, 64 were from Facebook, 57 were from YouTube, and 16 were from Twitter.

The following key themes emerged through analysis of Indonesian social media users’ comments: disbelief in the existence of acute ASHUE; suspicion about a potential new business after COVID-19; suspicion of COVID-19 vaccine(s) as the cause of ASHUE; religion-related fatalism; and trust in government measures. See Table 2 for an explanation of each theme, which we evidence with quotes throughout the remainder of this section. All comments are reported by theme rather than by type of social media.

**Table 2:** Summary of themes

Theme	Explanation	Number of posts
Disbelief in the existence of ASHUE • disciplining the public via power/control including fear tactics	Do not believe in the existence of the disease; believe in conspiracy theory; exaggerated news; believe this is a bioweapon; do not believe hepatitis occurs in children; and do not care about news on its preventive measures Apprehension with the word “mysterious”, a strategy for disciplining people to continue to follow COVID-19 protocols	38 12
Suspicion about a potential new business after COVID-19	Suspect of new medicine and vaccine business emerging that requires people to buy medicine and to be vaccinated	35
COVID-19 vaccine as the cause	COVID-19 vaccine as the main cause of ASHUE, and the disease occurs after mass vaccination of children	26
Religion-related fatalism	Trust more in religion or God instead of health experts and health logical perception	15
Trust in government measures	Social media users suggested public to follow COVID-19 protocols, asking the public to see the fact of mortality, and suggesting the government to keep informing the public about the disease and preventive measures	11

### Disbelief in the existence of ASHUE

The findings showed that social media users were aware of common hepatitis disease (types A, B, C, D and E) but they did not believe in the existence of new ASHUE and its severe impacts. Comments suggest the public perceive the impacts of ASHUE were not as serious as conveyed by the Indonesian Government via social media posts. They also suggest that COVID-19 (SARS-COV-2) was used as a barometer of disease severity. The following excerpts from our social media analysis reflect such perceptions:

*“I do not believe in the existence of ASHUE because hepatitis has been for a long time, which is known as yellow disease. There are only hepatitis A, B, C, D, and E. That’s it. It is impossible there is new hepatitis called mysterious” (User #34).*

*“Do not exaggerate this disease with the word mysterious. Hepatitis (common types) exists but its impact is not as bad as coronavirus” (User #98).*

Some social media users underlined their experiences or their family members’ experiences of recovering from hepatitis (*A, B, C, D, and E*), which they seemed to use in order to caution others’ belief in the existence of ASHUE and its severity:

*“I suffered from hepatitis since I was 20 years old and the doctor said I had only 3 months to live. When my first son was 1 year old, the doctor said to me that I had only 6 months to live. Now, I am 47 years old and I am healthy. Please do not easily believe the news about* ASHUE*” (User #45).*

*“My kid was sick because of hepatitis but now he is healthy, thanks God. This is only an example. Many people recovered from hepatitis… My uncle suffered from hepatitis but then he recovered” (User #135).*

Limited knowledge about how hepatitis is transmitted and a lack of data or evidence about the number of ASHUE cases in adults appeared to influence public trust in the validity of information on social media about the disease and its existence. For example, some users’ limited understanding of sexual contact as the only means of hepatitis transmission appeared to lead them to doubt the validity of information about ASHUE cases occurring in children. Similarly, the Indonesian Government’s information about the possibility of disease transmission in adults without evidence fostered disbelief among the users about its validity. The following quotes from two social media users highlight the limited knowledge and doubts people had about ASHUE and its existence:

*“It is impossible to have hepatitis in kids or children because hepatitis transmits through sexual intercourse and exchanging saliva. Kids do not do this. Kids only play” (User #19).*

*“In the beginning the government said the disease (*ASHUE*) commonly attacked children, but now government said it might also occur on adults. In fact, there is no evidence about adults” (User #12).*

#### Disciplining the public via power/control including fear tactics

Within social media users’ comments on Government advice disseminated through social media, the emergence of ASHUE was linked to concerns around public compliance to non-pharmaceutical prevention protocols for COVID-19. Many contended that the use of the word “mysterious” by the Government aimed to scare the public into being more obedient to COVID-19 prevention protocols, as illustrated in the following posts:

*“Because people do not care about COVID-19 anymore, the government starts making new issues to scare the public to keep wearing masks” (User #4).*

*“The government tends to scare people. We already know hepatitis but now the government scared us with the word mysterious” (User #29).*

Furthermore, through social media users’ comments on Government social media posts, it seemed that the public felt ASHUE was deliberately created and spread. Along with SARS-COV-2 responsible for COVID-19, this new disease was perceived as part of a conspiracy to control the global population. Social media users appeared suspicious and accusatory of the Indonesian Government and media, suggesting they overstated risk by framing the disease with the word “mysterious”. Highlighting these views, social media users wrote:

*“Coronavirus almost ends and now government creates a new issue with ASHUE. This is media framing, so please be smart to easily not trust news on acute hepatitis” (User #8).*

*“This is part of the global plan to reduce the population size globally. Many people died due to COVID-19 and it almost ends. Now, they create another disease called mysterious hepatitis” (User #33).*

*“After coronavirus, now they created hepatitis virus. We do not know what kind of virus after this one. It is a public secret to reduce the population size” (User #74).*

Social media users who did not believe in the existence and serious impacts of the ASHUE described indifference towards the Government’s recommendation of preventive measures and seemed to use their healthy condition as a barometer to not comply with preventive measures. As users wrote:

*“I do not believe it exist and I do not really care with this news or this disease (ASHUE). I do not want to listen to what government said about this disease and its preventive measures” (User #5)*

*“I do not care about the mysterious disease (ASHUE). The proof is that I am not vaccinated and I am still fine. I never suffered from COVID-19” (User #15)*

#### Suspicion about a potential new business after COVID-19

Social media users conveyed suspicion about ASHUE, and linked its emergence with business potential for the Indonesian Government and businesses comprising either government officials or affiliated with government officials, which could gain capital from such a disease. Medicines/pharmaceuticals and the potential for new vaccines, and protective masks are among products suspected to provide business and capital raising opportunity. Social media comments conveyed public suspicions that the Indonesian Government and business could make huge profits, likening ASHUE to the COVID-19 pandemic which required people to consistently take medicines, get vaccinated, and wear masks as illustrated in the following quotes:

*“After COVID-19, now the new disease (ASHUE) appears. This will be a new business for medicine and vaccines for government officials. We wait what disease comes after this one” (User #39).*

*“I think the end of this news is selling vaccines by requiring every child to be vaccinated. The vaccine is not free. Every disease will be an opportunity for government and businessmen to easily gain money” (User #52).*

*“Too much news about this acute hepatitis. All TV channels and online news reported about this disease but in the end, sooner or later, people will be asked to wear masks or to be vaccinated… the more masks or vaccine people need, the more money government or businessman gains” (User #81).*

Previous corruption of social assistance funds for people affected by COVID-19 in Indonesia was a strong supporting factor for public concerns about the interference of business and commerce within ASHUE. Public suspicion of corruption and business potential seemed strong as the previous corruption case was committed by high-level government officials within the Indonesian Ministry of Social Affairs, and this seemed to influence public perceptions about the new ASHUE outbreak; social media users wrote:

*“Remember the corruption of social assistance funding by government officials in the Ministry of Social Affairs during COVID-19. Remember certain people become instantly rich from this fake rapid test kits. Affected children might get social assistance fund but they will not receive full assistance fund” (User #133).*

*“We can see which companies (owned by/affiliated with government officials) that produce masks and vaccines or imports vaccines from other countries. All about business at the end of this new disease” (User #142).*

Some social media users also expressed fears about restrictions in contact with others in order to prevent the spread of ASHUE. Such fear seemed more pronounced, given people were already experiencing COVID-19 restrictions and we got a sense that their fear was that the new ASHUE outbreak might worsen people’s existing economic hardships. Highlighting this point, users wrote:

*“We suffered due to COVID restriction. We cannot work. Our income decreased significantly. We do not want any new restriction” (User #51)*

*“We are tired and bored with the restriction related to covid-19. If there is a new restriction, we cannot work because workplace might be closed again” (User #71)*

#### Suspicion of COVID-19 vaccine as the cause of ASHUE

Suspicion of the cause of the emergence of ASHUE disease was another topic that aroused debate among social media users. Some users argued that the COVID-19 vaccine was the cause of ASHUE. The sequence of events and children becoming disabled after mass vaccination seemed to inform users’ negative perception of COVID-19 vaccines as the cause of ASHUE. Some appeared to argue that they never heard of the disease before children were required to be vaccinated for COVID-19, and this seemed to increase feelings of vulnerability to the newly publicized ASHUE. Highlighting these notions, users wrote:

*“I think mysterious hepatitis (ASHUE) comes from the previous vaccines (COVID-19 vaccines). According to the news many were paralyzed and died after being vaccinated” (User #2).*

*“I am disappointed to be vaccinated. I am more susceptible to hepatitis after being vaccinated…The fact that the disease (mysterious hepatitis) emerged after mass vaccines to children” (User #41).*

*“This is because there is no laboratory research on the content of AstraZeneca and Moderna vaccines before they are injected into the human body. As a result, children have to suffer from this acute hepatitis” (User #5).*

Suspicion of the COVID-19 vaccine as causing ASHUE in children also seemed to be influenced by users’ misperception about the ingredients of the vaccine. Users from the Islamic religion perceived that the ingredients of the COVID-19 vaccines are derived from pork, which is haram and believed to negatively affect children’s immune system, for example:

*“This is because of the (COVID-19) vaccines. The vaccines are from pig which is haram (in Islam). Anything from haram will create a new problem for health” (User #37).*

*“Do not force children to be vaccinated. Vaccines from pig affected their immune system. Children already have an immune system from God. We just keep their immune system” (User #71).*

*“If the vaccine is not from pig, it won’t create problems like today” (User #95)*

Furthermore, suspicion of COVID-19 vaccines as the cause of ASHUE and misperception about the ingredients of the vaccines seemed to have negative implications on users’ attitudes and behaviors towards the ASHUE vaccine. Many users underlined that they did not want to be vaccinated against the new hepatitis virus and did not want to be an ‘experimental’ body for the vaccine. In addition, the experience of the ineffectiveness of the COVID-19 vaccines seemed to also influence their decision to not get vaccinated for ASHUE. These concerns are reflected in the following comments:

“*I am 40 years old now. I have not been vaccinated but I am okay (healthy). One of my friends, the same age as me, has been hospitalized several times although he has been vaccinated. If we are asked to have a vaccine for this acute hepatitis, I do not want” (User #47).*

*“After the initial COVID-19 variant, then delta and then Omicron variant emerged… We (refer to people in Indonesia in general) have been vaccinated 3 times. We do not know what the next vaccine to fight this current disease (mysterious hepatitis) is. I do not want to be vaccinated again. I do not want my body to become a place for a trial” (User #28).*

However, some social media users’ comments refuted previous arguments about the suspicion of the cause of ASHUE and misperception about the ingredients of the COVID-19 vaccine and suggested that the public felt there was no correlation between ASHUE and the COVID-19 vaccine. They also argued that the public need to read less negative and science-based news sources from health professionals:

*“There is no evidence of the correlation between mysterious hepatitis (ASHUE) and COVID-19 but just coincidence” (User #27).*

*“This is not related with COVID-19 vaccine as children suffered from the acute hepatitis were not vaccinated. Please read scientific information and listen to health experts” (User #11).*

#### Religion-related fatalism

Religious beliefs seemed to affect users’ perspectives on the disease, life, and death. Many social media users seemed to believe that God controls life and death and therefore, there is no need to be afraid of ASHUE or take actions to prevent it. Some users also highlighted their belief in God’s mercy to heal people from sickness instead of becoming knowledgeable of disease prevention. Some quotes below described such fatalism:

*“Death is from Allah. We can see people are fine although they are infected with COVID-19. Only God knows our death” (User #91).*

*“Do not worry with the disease (ASHUE), only fear Allah…I am not afraid of the disease (ASHUE). Life is managed by Allah” (User #49).*

*“God is merciful and He will heal kids who are sick. Do not worry, stop reading and sharing this news, remember God” (User #120).*

However, some social media users had different views about faith and health. They seemed to argue that having faith in God should lead to following health protocols in order to prevent the further spread of the disease as described below:

*“Having faith is not only with the mouth but also with the act of staying healthy, away from bacteria or viruses. Living and death are in God’s will, but human beings are given mind to think about how to prevent themselves from being infected by following the recommendations from health experts or government” (User #110).*

*“Being infected with the virus due to not wearing masks and then died…It’s your own fault, not because God wants you to die” (User #117).*

#### Trust in government measures

Some social media users posted positive comments in support of the material posted by the Indonesian Government; arguing that the Government played an important role in reducing disease incidence by disseminating news on preventive actions disease etiology. The following posts from some users show that they positively valued actions taken and information provided by the Government, as these helped them protect themselves and their children:

*“This news is important so that we can control our hygiene, wash hands with soap, avoid using tools together and avoid children having contact with sick people. If we do this, we save our life. The government has done a good job to make people aware of the danger of the disease” (User #23).*

*“The government needs to keep disseminating such news to prevent further impacts on children and people in general… The government needs to seriously handle this disease and disseminate its protocol to prevent the spread of the disease” (User # 37).*

Another support from social media users to the Indonesian Government was reflected in suggestions for laboratory research action to understand the disease instead of merely following the information that other countries or the WHO provided about the disease. Social media users suggested to the Government the need for investment in health research, laboratory facilities and human resources in the health domain as presented in these quotes:

*“Indonesia needs to conduct further laboratory research about the disease. Do not just follow what other countries said” (User #30).*

*“The government needs to provide good laboratory facilities and qualified laboratory workers in several provinces to face such outbreak in the future” (User #58).*

## Discussion

This study is the first to explore Indonesian social media users’ perceptions (via their personal comments made on social media) about the emergence of ASHUE, and their attitudes towards preventive measures recommended by the Indonesian Government. This illuminated public perceptions of the efficacy of disease countermeasures. Since the WHO declared the ASHUE outbreak in April 2022 [1], there has been a very limited number of studies to provide evidence of public perceptions [5, 8]. The findings presented here show the relevance of several key themes that warrant consideration if Indonesian Government disease messaging is to be effective including disbelief in the existence of ASHUE (exaggerated news, conspiracy, indifference), suspicion about a potential new business after COVID-19, suspicion to COVID-19 vaccines as the cause, religion-related fatalism, and trust in government countermeasures.

Our findings suggest that the majority of opposing comments towards Government social media posts about the emergence of ASHUE and its serious impacts, result from disbelief about the emergence of ASHUE. At a superficial level, this might be explained by the limited awareness of disease causation among Indonesians. Evidence suggests the public has very limited knowledge and misperceptions about ASHUE and its means of transmission [16, 17]. This potentially draws false conclusions about ASHUE, as simply media framing (referring to ASHUE as ‘mysterious’) and fear tactics employed by the Government designed to discipline and control people. These findings are in line with previous studies on conspiratorial understandings during the COVID-19 pandemic which expose perceptions that COVID-19 was part of global bioweapon program to control the population [18, 19]. A more in-depth view of our findings reveal public perceptions that the Indonesian Government is underprepared in terms of resources that structure its health system, to capably respond to ASHUE, while it is already managing COVID-19. It could also be said that due to its unknown etiology, the Government seems to be facing a challenge to provide tailored prevention and control measures for ASHUE. Perhaps a consequence of the lack of knowledge about and disbelief in the existence of ASHUE and its severe impacts, the ‘mysteriousness’ of the disease which means social media users (which we take to represent the public) will ignore or not comply with preventive measures recommended by the Government. This is in accordance with the concept of perceived susceptibility and severity and suggests that individual decisions to respond to recommended preventive behavioral measures is determined by people’s level of knowledge about how susceptible they are to an infection or disease and their perceptions of the seriousness of the impacts of the infection or disease on their health [20–22].

Our findings suggest a worrying element of public distrust in the Indonesian Government and its motivations; with important negative consequences for public adherence to disease prevention advice disseminated on social media. The reality of massive corruption in Indonesia, including the current corruption of COVID-19 funds and other social assistance funds by Government officials and their business allies or companies [23, 24] has potential to cultivate negative public perceptions about the Government generally, and reduced public trust in the Government’s work including disease prevention measures. As identified in social media users’ posts analyzed, the perception that ASHUE was ‘deliberately created’ by the Government for business and capital-gains purposes is an example of distrust and negative public perception about the government, which in turn inhibits public adherence with preventive measures. The public’s suspicion about the possible business agenda contained in this ‘created’ ASHUE issue seems to be based on the facts regarding the involvement of the Indonesian ministers through their companies in COVID-19-related businesses, including in the provision of polymerase chain reaction (PCR) test, COVID-19 drugs and vaccines, as well as the provision of counterfeit COVID-19 Rapid Antigen test kits in Indonesia [25–27]. Thus, it is plausible to argue that public suspicion of corruption seems to be stronger than is the awareness of the risks of being infected and affected by ASHUE and its severe impacts. Suspicion of a new business agenda after ASHUE seems to lead to further fear of possible new restrictions or new protocols that might affect individual mobilities. Such fear is understandable as many people’s economic life has been already adversely affected by restrictions related to the COVID-19 pandemic.

Our analysis also exposes the influence of religious beliefs on the views of social media users about ASHUE, reflected in the suspicion that COVID-19 vaccines contain pork ingredients which are considered *haram* in Islam. This led to the claim that (and the blame directed towards) COVID-19 vaccines as the causes of ASHUE, and to deaths and disability among children following COVID-19 vaccination. Such claim and blame by social media users seemed to be influenced by media exposure about Indonesian children’s deaths or becoming disable after receiving COVID-19 vaccines which is in line with a study reported elsewhere [28]. Religious beliefs underlying users’ suspicion and claim is that anything comes from *haram* things, including ingredients, foods, actions, could create further health problems or serious complications and cause people to fear that if they are vaccinated, they will go to hell [29, 30]. There is a future risk of public rejection of ASHUE vaccines. Our findings here are congruent with the concept of healthcare acceptability in the Access to Healthcare Framework, which suggests that the acceptance of a healthcare service is determined by how well it aligns with sociocultural norms and values, and religious beliefs of communities where the service is being delivered [31–33]. This study also highlights faith-related fatalism among social media users who underlined the importance of fearing God’s punishment instead of compliance with preventive measures or biomedical perception of risks. This supports the findings some studies in Iran and Pakistan, where Muslim participants believed that life and death are managed by God, and that human’s efforts do not affect the moment of death [34, 35], which then affects their perceptions of health concerns [36], leading to hesitancy to adopt or non-compliance with COVID-19 preventive protocols. In addition, this study reported here also shows public support for the Indonesian Government to capably respond to the emergence of ASHUE through investment in or provision of sufficient healthcare facilities and healthcare professionals. Although supporting the Government was the least posts and debates identified in the three social media platforms, it is very important as an early response to a disease outbreak may lead to better management of the disease and help prevent deaths and reduce economic costs [37, 38].

### Strengths and limitations of the study

Extracting data from three different social media platforms is a strength of this study because each social media platform is used by different population subcultures, and thus there is increased opportunity to capture variability in public perceptions [39]. Rather than considering people’s comments collected and analyzed in this study as objective and discrete ‘social facts’, they are considered to be intertwined with dynamic social, cultural and political meanings from which they cannot be separated [40, 41]. This helps to account for the impossibility of firm boundaries within qualitative social media research, where research settings rarely have firm physical boundaries but unfold haphazardly in a global virtual environment [42]. As such, the analysis proceeded according to units of meaning – rather than a uniform and unvarying standard around procedural concerns like how much text to code [43]. Having multiple authors involved in data collection and coding supported reflexivity amidst data analysis and interpretation through discussion of similarities, disciplinary viewpoints and being cognizant of individual perceptions.

To the best of our knowledge, this is the first study examining public perceptions of ASHUE using qualitative analysis of Indonesian Government social media posts and thus offers crucial insight relevant to disease response measures. However, the study has some limitations. Three social media platforms were used as data sources, thus perceptions of social media users in other social media forums are not covered. The characteristics of the users who posted on social media were not available, hence authors cannot include sociodemographic and economic profiles of the users. Regardless, they showcase publicly available and circulating information about ASHUE.

## Conclusions

This paper has provided insight into public perceptions about Government-led prevention messaging on social media following the emergence of ASHUE and has discussed implications for how perceptions might influence their behaviors toward evidence-based preventive measures, with implications for curtailing disease spread. Findings demonstrated that concern about disbelief in the existence of ASHUE, skepticism about potential new business related to vaccine development, causal links between COVID-19 vaccines were significant issues. The findings also showed that social media users believed more in religion or God than logical health perception. Additionally, we found positive supports for the efficacy of government-led preventive measures. Understanding public perceptions about this outbreak is useful to inform future Government practices and to develop programs and activities that can help enhance public awareness about ASHUE and its possible consequences, and the available healthcare support they can access if infected. The findings of the study highlight the practical implications for Indonesian Government and public health agencies to improve their level of responses to the public health problems and information dissemination through their official media platforms and to ensure health system is ready to face future outbreaks [44].

## Data Availability

The dataset supporting the conclusions of this article is included within the article.
